# A computational approach for perturbation-induced EMT transitions

**DOI:** 10.1038/s41540-025-00597-9

**Published:** 2025-11-13

**Authors:** Daniel Ramirez, David A. Kessler, Mingyang Lu, Herbert Levine

**Affiliations:** 1 Department of Bioengineering, Northeastern University, Boston, MA USA; 2 Center for Theoretical Biological Physics, Northeastern University, Boston, MA USA; 3 Department of Physics, Bar-Ilan University, Ramat-Gan, Israel; 4 Department of Physics, Northeastern University, Boston, MA USA

**Keywords:** Dynamical systems, Multistability, Regulatory networks, Stochastic modelling

## Abstract

The Epithelial-mesenchymal transition (EMT) is a cellular state transition fundamental to development, wound healing, and cancer metastasis. The gene regulatory mechanisms underlying EMT have been extensively documented, revealing gene regulatory networks (GRNs) involving groups of mutually inhibiting transcription factors and microRNAs. Despite significant progress from both experimental and computational approaches, the details of how the EMT GRN initiates EMT in response to various external inputs is still not well understood. Here, we apply both Boolean and ordinary differential equation (ODE)-based methods to simulate a well-studied 26-node, 100-edge EMT GRN, examining its response to a wide range of single- and double-node perturbations. We evaluate the characteristics of effective EMT-inducing signals, particularly examining the amplifying role of transcriptional noise in determining the likelihood and mean transit time of an EMT. Together, these models establish a complementary framework for understanding and predicting drivers of EMT in the context of a GRN. We anticipate that this framework for a systematic study of in-silico GRN perturbations will be useful in developing increasingly accurate dynamical GRN models for various biological processes.

## Introduction

GRN inference and Modeling has been applied in recent years to describe the phenotypic distribution of a biological system, uncover key transcription factors (TFs), and predict perturbation responses^1,2^. In the case of EMT, GRN models have been constructed which recapitulate epithelial (E), mesenchymal (M), and intermediate gene expression states, revealing multistability across a wide range of parameters^3–5^. However, understanding the dynamic effects of a perturbation from a GRN model in a time-resolved manner remains an open challenge. The ability to do so could guide future experimental study of EMT, as well as numerous other biological systems described by a GRN. Furthermore, it is believed that transcriptional noise and cell-to-cell variability^6^ may play important roles in determining cell fate, but these features are excluded from some GRN models, which tend to describe tissue-level behavior.

Computational modeling using a variety of approaches provides a useful framework to address these challenges. Boolean modeling is a much-used approach^7–10^ which abstracts gene expression into binary states and has been used to uncover key regulators and network structures necessary to produce the observed dynamics. Boolean models are particularly effective at capturing multistability, particularly in large networks which may be difficult to accurately parameterize. On the other hand, ordinary differential equation (ODE) based methods with its continuous numerical tracking of GRN states, enables a higher-resolution study of quantitative and temporal variation in EMT trajectories. Stochastic differential equations (SDEs) also permit greater flexibility in modeling noise in biological processes, which is known to have a major influence on the dynamics of transcriptional regulation^11^.

In this study, we present an integrated analysis of a specific EMT GRN using both Boolean and ODE-based strategies to systematically identify and characterize the perturbations most conducive to inducing an EMT. Beginning with the Boolean framework, we identify critical nodes the clamping of which effectively and irreversibly drive initial E-type states to an M-type state. We perform simulations at multiple levels of transcriptional noise, modeled via a pseudo-temperature, finding that noise amplifies EMT signals, but transitions can be achieved even at very low noise with an optimal signal. Complementing the Boolean model, we applied RACIPE, an ensemble ODE-based GRN simulator, to the same EMT GRN to generate an ensemble of bistable models and simulate the dynamics under various signals and levels of transcriptional noise. These continuous simulations reveal general agreement between the two approaches as to the efficacy of various signals, while incorporating a degree of variability between model parameter sets. This combined approach demonstrates how intrinsic heterogeneity and external applied noise jointly influence the nature of the EMT in response to a given external perturbing signal.

## Results

**Fig. 1:**
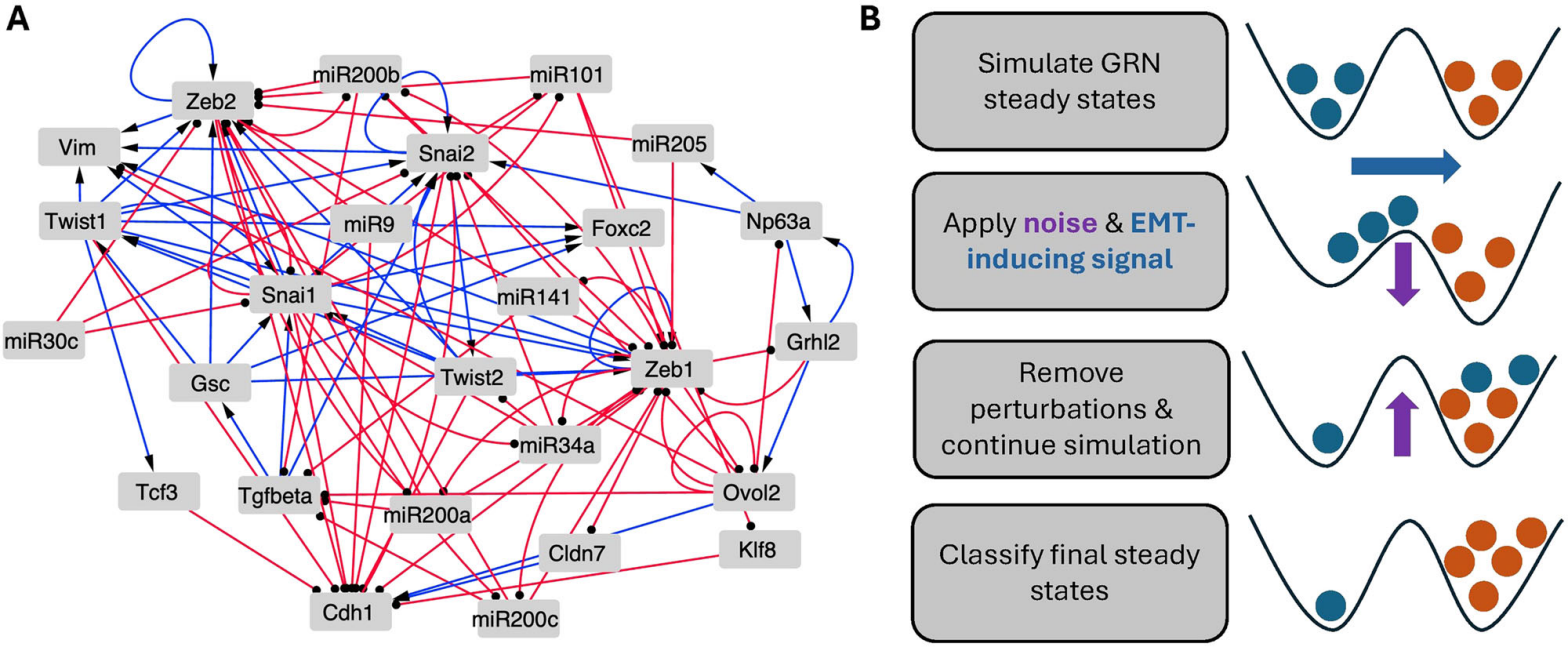
The EMT transcriptional network and simulated perturbation approach. **A** 26-node, 100-edge EMT transcriptional GRN constructed from published experimental evidence. Nodes correspond to TFs or miRNAs, blue arrows represent activation, and red round-tipped arrows represent inhibition. **B** A diagram of our simulated EMT-inducing perturbation approach. E and M steady states are first obtained from random initial conditions, then a transient driving signal alongside noise is applied. Both noise and signal are removed and the simulation is further evolved to obtain the final state.

We study an EMT regulatory network which was obtained from a previous work^12^, and has 26 nodes and 100 edges (Fig. 1A). The unperturbed dynamics in both the Boolean and RACIPE frameworks have been studied in previous works^5,12–14^, and the GRN displays a very large set of stable states with both epithelial (E) and mesenchymal (M) type states represented. In the Boolean model, these E- and M-type states were characterized by the property of low frustration^14–17^, i.e., one in which most links in the network were satisfied. In the RACIPE class of models, the dynamics was seen to result in an essentially one-dimensional state space, with the E and M type states found at the two extremes. In our current study, simulations from random initial conditions were used to first obtain a set of stable E states which were taken as the initial conditions for our perturbation simulations. Perturbations were modeled by clamping one or more network nodes (to a binary value in the Boolean model, or a value associated with the target steady state in RACIPE) and simulating the dynamics for all other nodes for some duration. (See SI for results regarding other implementations of perturbations which we explored.) Finally, the signal was removed to obtain the new post-perturbation steady states (see Methods). A successful EMT was defined as a run in which the transient signal induced an irreversible transition from the initial E type state to an M type state (Fig. 1B).

In general, one might expect transitions in a bistable system to take one of two forms. In the first, a signal changes the landscape such that the initial stable state either goes unstable or ceases to exist altogether, causing cells to deterministically proceed to the other remaining state. Such transitions might be expected to require a strong signal, sufficient to destabilize or destroy the initial state. On the other hand, a weaker signal could drive EMT if accompanied by sufficient transcriptional noise to traverse the barrier between states, while leaving the initial steady state modified but intact. This method might be more effective in driving a heterogeneous cell population where the signal may otherwise have variable effects. Aiming to uncover the properties of effective EMT-inducing signals, we simulated a wide range of transient perturbations. To evaluate the interplay of EMT signals and transcriptional noise, we compared deterministic simulations with those where varying levels of transient noise are applied during the signaling period.

### Boolean network modeling characterizes EMT signal efficacy

Boolean models of EMT have been extensively explored by several groups^5,7,8,14^. These models are constructed from biological data regarding the sign of the various influences on gene expression levels and typically use a simple version of a majority rule whenever multiple regulatory links impinge on a particular node. Full details are given in the Methods section. An important finding^14^ is that, in general, these models exhibit very many possible steady states, but almost of all these have very shallow basins of attraction and are very unstable in the presence of finite amplitude noise. The remaining states are either strongly E-like, i.e., have high expression of most epithelial genes and low expression of mesenchymal ones) or strongly M-like. These stable states, in contrast to the (relatively) unstable states, were found to be associated with an extremely low degree of frustration in these states, namely a quite small number of regulatory links that are “violated” by the steady-state node values. Here, we study the question of what types of perturbations can cause transitions between these two classes (E/M) of low-frustration states in the aforementioned 26 node EMT network (node name mappings for the Boolean model are given in Table S1).

**Fig. 2:**
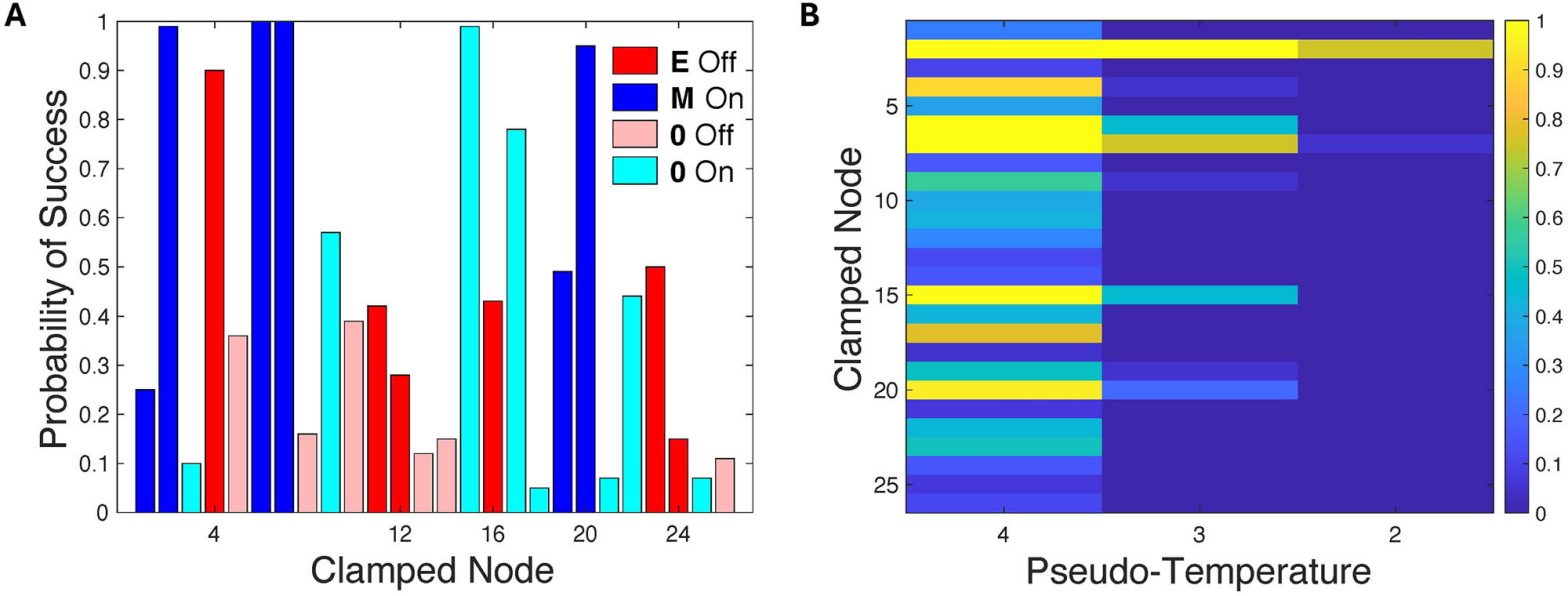
Efficacy of single-node perturbations in the Boolean EMT GRN model. **A** The percentage of successful transition for each node. $T=4$, ${t}_{c}=400$. The nodes with epithelial score 1 are clamped off and colored dark red. Those with epithelial score -1 are clamped on and colored dark blue. Those with score 0 that are more effective in inducing a transition when clamped off are colored cyan while those that are more effective when clamped on are colored pink. **B** The percentage of successful transitions for each node. $T=\mathrm{4,3,2}$, ${t}_{c}=400$. Node numbers are mapped to gene names in Table S1.

We generated random initial conditions and simulated the system until it reached a low-frustration state, then scored these states as either E or M based on prior gene associations (see Methods). The E states were used as starting conditions (due to the symmetry of the Boolean model, the backward MET transition has the exact same properties) for the perturbation simulations, where various small sets of selected nodes were clamped to the target value and the simulation continued with pseudo-temperature $T$ for duration ${t}_{c}$, before a final simulation phase where the clamps and noise were removed, with final states being similarly designated E or M (see Methods). For genes not associated with E or M in our scoring system, both clamp values were simulated, and the one leading to a higher percentage of successful transitions was included in our tabulations. We began by performing multiple series of runs, where in each series of 100 runs a single node was chosen to be clamped. The percentage of successful transitions for each node (using $T=4$ and ${t}_{C}\,=\,400$) is presented in Fig. 2A. We see that there is a wide range of transition probabilities for the various node choices, but overall, clamping E nodes off was less effective than clamping M nodes on. To explore further, we repeated the experiment for lower temperatures. The results are presented in Fig. 2B. We see that clamping node 2 (Zeb1), a mesenchymal node, to the on state is the most effective intervention. Nevertheless, we see that at ${T}=\,2$ the success rate is beginning to be impacted, and by ${T}=\,1$ (data not shown) it is less than 1%, even for Zeb1. Increasing ${t}_{c}$, however, has the effect of increasing the success rate. For example, for *t*_*c*_ = 1600, the success rate for clamping Zeb1 on at a noise level of ${T}=\,1$ is 4%, at *t*_*c*_ = 6400 it is 22% and by *t*_*c*_ = 25600 it is 58%. This demonstrates that there is clearly a finite pseudo-energy barrier between E and M states even with node 2 clamped.

**Fig. 3:**
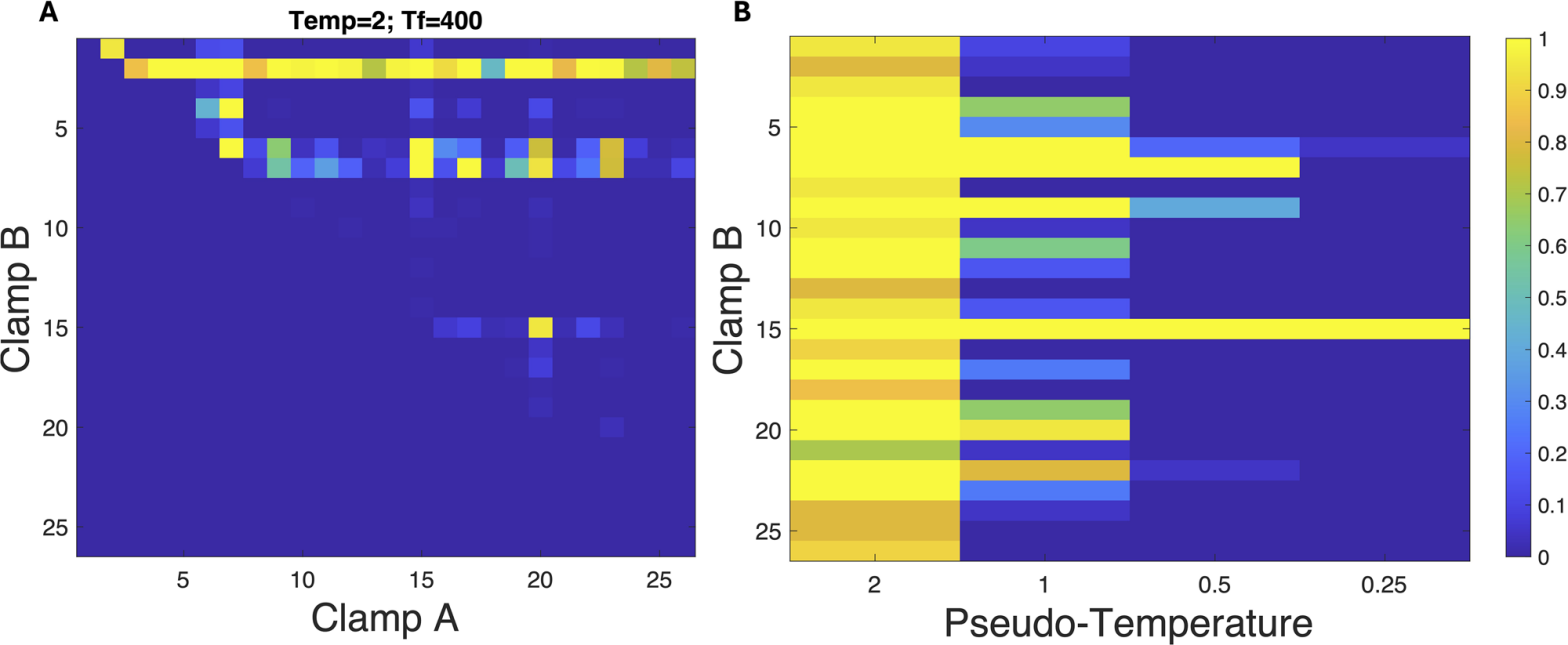
Zeb1 is most effective in driving EMT and potentiated by Snai2. **A** The percentage of successful transition for each pair of node clampings (A > B). $T=2$, ${t}_{c}=400$. **B** The probability of successful transition for clamping node 2 on along with one other node. $T=\mathrm{2,1,0.5,0.25}$; ${t}_{c}=400$.

An obvious next question is whether clamping two nodes removes the pseudo-energy barrier completely. To investigate this, we clamp all pairs of nodes, either on or off as proved most efficacious in the single node clamping experiments. The results for $T=2$ are shown in Fig. 3A. Unsurprisingly, having node 2 as one of the clamped nodes is clearly superior. To proceed, we clamp node 2 on and then run over all the other possibilities for clamping a second node for various values of $T$. These results are shown in Fig. 3B. Clearly, node 15 (Snai2) on is the best choice for the second clamp, and here the transition is deterministic, as it persists to the smallest noise level that we have explored.

### RACIPE: variations on the theme

**Fig. 4:**
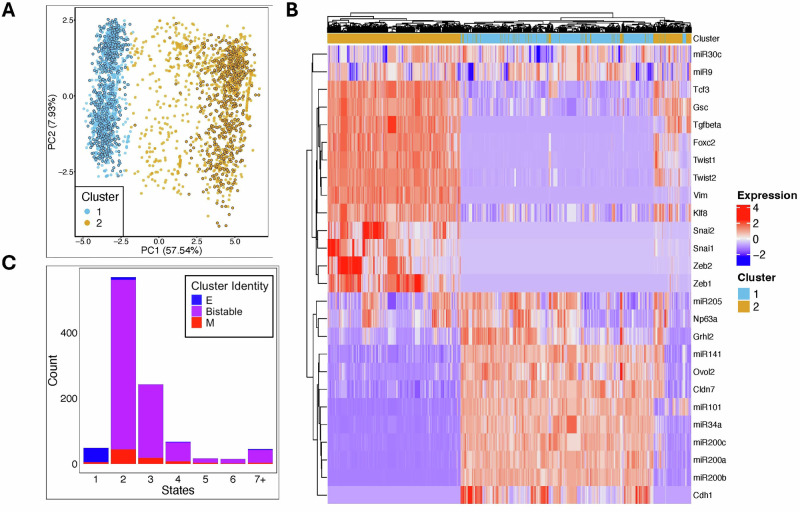
The EMT transcriptional network reflects observed phenotypes and promotes bi-stability. **A** PCA projection of the unique steady states obtained from simulated models of the EMT GRN, colored according to labels from Gaussian mixture model clustering. Points with black outline represent states from bistable models selected for further analysis. **B** Heatmap of gene expression levels across the EMT GRN steady state distribution, annotated on top with cluster labels. **C** Histogram of the number of unique steady states per model with respect to clusters is shown, indicating that the vast majority of models are multi-stable with steady states in both E and M. clusters.

We wanted to determine the extent to which the results given above depended on our detailed simulation protocol (and in particular the Booleanization of expression levels) as opposed to just depending on the network topology. To do this, we employed RACIPE, an algorithm which takes as input a GRN and creates an ensemble of ordinary differential equation (ODE) models with randomized parameters (see Methods). Simulating this ensemble of models, each generates one or more stable steady states. We found two large clusters of steady-states (Supplementary Fig. 1) which can be classified by the first principal component of the expression matrix and conform to prior knowledge of key EMT regulators (Fig. 4A, Supplementary Fig. 2). One cluster is characterized by higher average expression of nodes commonly associated with the epithelial (E) state, such as miR34, miR101, miR200, and Cdh1^18–20^; the other cluster is marked by higher expression of several mesenchymal (M)-associated genes, such as Twist2, Twist1, Vim, Foxc2, and TGF-$\beta$^18^ (Fig. 4B). The unique steady states slightly favor the E state, with 59% E and 41% M.

Previous studies of the RACIPE algorithm on the 26-node EMT GRN focused on the properties of the collection of all steady-states, as above. For our purposes, however, as we are interested in transitions between E and M states, it is not sufficient that the ensemble of models includes both stable E and M states, but rather it is critical that each given model possess both stable E and M states. To investigate this point, we simulated 1000 randomly parameterized models, starting from 200 randomized initial conditions each. The resulting steady states were then aggregated to estimate the number of monostable and multistable models. Notably, the vast majority of models constructed from randomized parameter sampling were multi-stable, and more specifically, bistable such that there were at least two stable states with at least one each belonging to the E and M clusters (“E/M bistable”) (Fig. 4C). A subset of 500 E/M bistable models was selected for perturbation simulations to match the bimodal structure of the low-frustration stable states in the Boolean model (black outlined points in Fig. 4A).

To simulate EMT induction, we started the ensemble system at steady state (initializing each of the 500 bistable models in its E-state, see Fig. 4A) and considered which perturbations caused a high proportion of E-states to transition towards the target M-states. To identify effective EMT driver perturbations, we simulated the ensemble of models as in the Boolean case with 351 different signals, the 26 single node perturbations (via clamping) and the $$325 =\left(\begin{array}{c}{26}\\{2}\end{array}\right)$$ double node perturbations, either under deterministic or stochastic conditions. Perturbations involved fixing the value of the clamped gene(s) at their M-state values while simulating the dynamics for other nodes normally. To quantify the effect of transcriptional noise on the likelihood of spontaneous transitions, we first performed deterministic simulations of a signal-driven EMT as a baseline reference point. Of the library of 351 signals, the vast majority were largely ineffective in deterministic conditions, with only 99 achieving a conversion rate above 5% and only 26 signals which drove more than half of the models through EMT (Supplementary Fig. 3A). Of the single-gene deterministic signals, the only one with a significant efficacy was Zeb1, driving 50% of E models to the M state, reflecting the heterogeneity of the structure of the phase space of the different models. Note that while Zeb1 was also the most effective signal in the Boolean model, there it was purely a noise-driven effect, with no effect in the deterministic limit. The most effective two-component deterministic signal was the combination of Zeb1 and Snai2, which led 87% of models to EMT. Zeb1 was a constituent component of all of the signals which had notable deterministic efficacy. Besides Zeb1, the other signals with substantively non-zero efficacy in the deterministic case included TGF-B, Snai1, Snai2, and Zeb2. Interestingly, the known EMT TFs Twist1 and Twist2 appeared to be weaker drivers of EMT.

Next, we simulated control cases with only transient noise, and no transcriptional signal, and evaluated the proportion of models which ended the simulation in a different state than they began. Noise was modeled as an Ornstein-Uhlenbeck process with correlation time 10 and multiple variance levels, aiming to increase the probability of transitions without eliminating the separate basins entirely (see Methods for details). We found that a sufficiently high noise level can drive models between E and M states at a high rate, with up to 20% of models undergoing EMT/MET at noise levels above 0.2. Interestingly, we found that the effect of this noise was not symmetric; at lower noise levels, MET was more prevalent, whereas with high noise, EMT was favored. (Supplementary Fig. 4A). Examining the tendency of models over repeated trials, we see that most models in the ensemble consistently favor one direction, with the preference becoming stronger at higher noise levels. This shows that the relative basin depth and stability of the E and M states is highly variable within the ensemble, with some models exhibiting similar numbers of EMTs and METs even at high noise (Supplementary Fig. 4B). Of course, the asymmetries revealed in some RACIPE models are not present in the Boolean model, which is symmetric by definition.

**Fig. 5:**
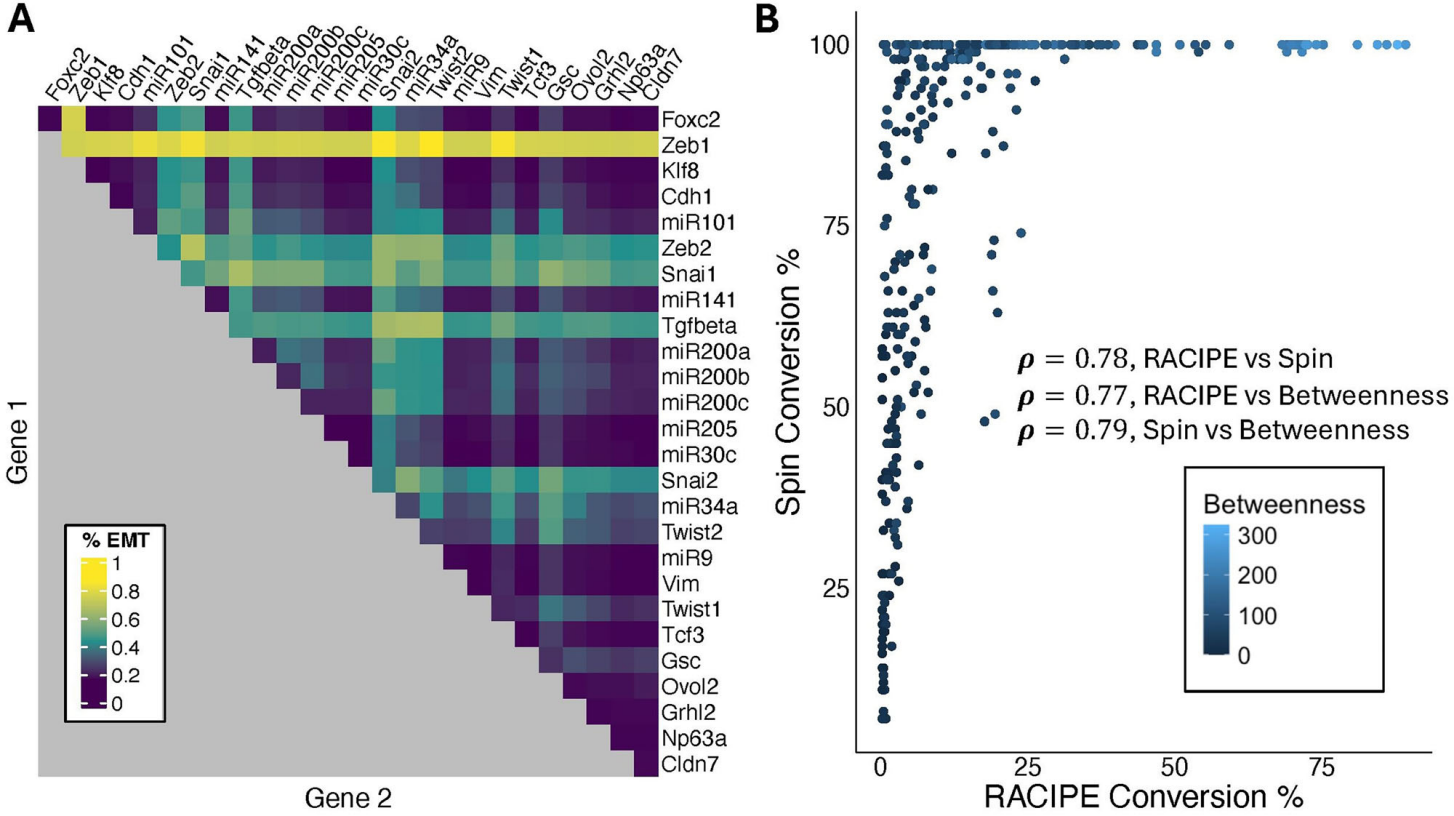
Properties of EMT-inducing perturbations in the ODE EMT GRN model. **A** Heatmap of simulation results for all 2-node perturbations with noise level 0.04. Rows and columns denote clamped gene combinations, (with the single node signals display along the diagonal) and the color shows the proportion of models which began in an E state and ended in an M state after transient signaling and noise followed by deterministic relaxation. **B** Scatterplot of signal efficacy represented by percentage of E models undergoing EMT in Boolean model vs RACIPE, colored by group betweenness centrality of perturbed nodes for each signal. Spearman correlation coefficients between RACIPE efficacy, Boolean efficacy, and betweenness centrality annotated on plot, suggesting betweenness centrality is a reasonable predictive measure of efficacy across modeling frameworks.

To create conditions where noise facilitated EMT-inducing signals but did not directly drive transitions, we selected a noise variance of 0.04, such that a very small fraction ( ~ 1%) of models underwent spontaneous EMT with noise but no signal. Our library of all 351 1- and 2-gene driving signals was then applied alongside this noise level. Again, the vast majority of signals were ineffective in driving EMT: the median signal efficacy over these 351 cases (i.e., the percentage of models beginning in an E-state and undergoing EMT) increased from 1.2% in deterministic simulations to 7.8% with the selected noise level, and only 32 signals achieved an EMT induction rate above 50%. Mirroring the deterministic results, Zeb1 was the strongest 1-gene signal and a component in many of the most effective 2-gene signals (Fig. 5A). The prevalence of Zeb1 and Snai2 among the most effective signals is consistent between the modeling approaches, but relative signal strength on the whole was not perfectly correlated (Fig. 5B, ${\boldsymbol{\rho }}={\boldsymbol{0}}{\boldsymbol{.}}{\boldsymbol{79}}$). For example, the Boolean model showed stronger EMT induction from Cdh1, Twist1 and Twist2 in general. Further increasing the noise level of the RACIPE simulations to a variance of 0.2 led to higher signal efficacies across the board, making additional one-gene signals such as Snai1/2, Zeb2, miR34a, and Twist2 viable EMT drivers (Supplementary Fig. 3B). The effect of noise on EMT conversion varied across signals, with some signals exhibiting a more pronounced increase than others (Supplementary Fig. 3C).

To evaluate whether the results from the 26-node network, for example the complementary effects of transition signals versus noise and the correlation between signal efficacy and betweenness centrality (see next section), were applicable to other GRN circuits, we applied the same workflow to another, larger EMT GRN. This new GRN contains 72 nodes and 142 edges and was obtained from ref. ^5^. Signaling simulations on the 72-node GRN showed that β-catenin, E-cadherin, and Snai1 were the strongest EMT-inducing signals, different in detail from what has been shown for the 26-node GRN. However, the relationship between centrality and signal efficacy was even stronger among the signals we simulated for the 72-node network, suggesting that this metric would be useful in identifying driver genes across different biological models (Supplementary Note, Supplementary Figs. 7-8).

Finally, we investigated the time for models to undergo EMT and its relationship to noise level. Using the signal where Zeb1 is clamped as an example, we ran 10 trial simulations and identified the point at which models first reached the M state, as defined by KNN clustering against the unperturbed states (see Methods). In general, most models transitioned by t = 100, with the cumulative number of transitions slowly increasing after this point. We found that increasing the noise level did not substantially change this pattern, but did increase the number of models able to undergo EMT (Supplementary Fig. 5). Given the long duration (t = 500) of our ensemble simulations, this resulted in very high consistency across trials, i.e. a model that underwent EMT in one trial was very likely to do so in every trial. As an aside, the positive relationship between clamp duration and number of completed EMTs is corroborated by experimental data: several studies have observed that sustained signaling increases the likelihood of irreversible EMT, whereas short-term exposure to an EMT-inducing signal can lead to rapid reversion to an epithelial state when the signal is removed^21^.

### The effectiveness of Zeb1 in both the Boolean and RACIPE cases

Given that Zeb1 was clearly the most effective single node signal in both the Boolean and RACIPE cases, it is reasonable to look to the network topology for the underlying explanation of this effectiveness. Overall, one clear feature of effective EMT-inducing signals was the total out-degree of the perturbed nodes. Zeb1 in this network has 11 out-going edges, more than any other node. Notably, Zeb1 also has a self-activating interaction which in particular may amplify its downstream effects and has a known link to the mesenchymal state^22^. Signal efficacy also correlated with group betweenness centrality, a metric of the perturbed nodes’ representation in shortest paths throughout the network (Fig. 5B, ${\boldsymbol{\rho }}={\boldsymbol{0}}{\boldsymbol{.}}{\boldsymbol{78}}$). Indeed, although Zeb1 has only one more outgoing edge than Snai1, it includes a self-activation link whereas Snai1 inhibits itself, and Zeb1 has a much higher betweenness centrality score of 218 compared to Snai1 with 67. In the noisy RACIPE experiments, signals involving Zeb1 were at minimum 72% effective with a median value of 75%, whereas signals involving Snai1 had a minimum efficacy of 23% and a median of 32%. In the Boolean model, on the other hand, signals involving Zeb1 and Snai1 all had efficacy of 99% or 100% at temperature $T=3$, though Zeb1 is more effective at lower T.

### A more detailed look at the dynamics of the transition in the two approaches

As shown in Fig. 5, the two modeling approaches showed strong agreement at the aggregate level in the ranking of effective and ineffective signals, although the Boolean model generally showed higher maximal percentages of models undergoing EMT. The Spearman correlation between EMT induction percentages across models suggests that the two modeling approaches encode similar high-level dynamics.

**Fig. 6:**
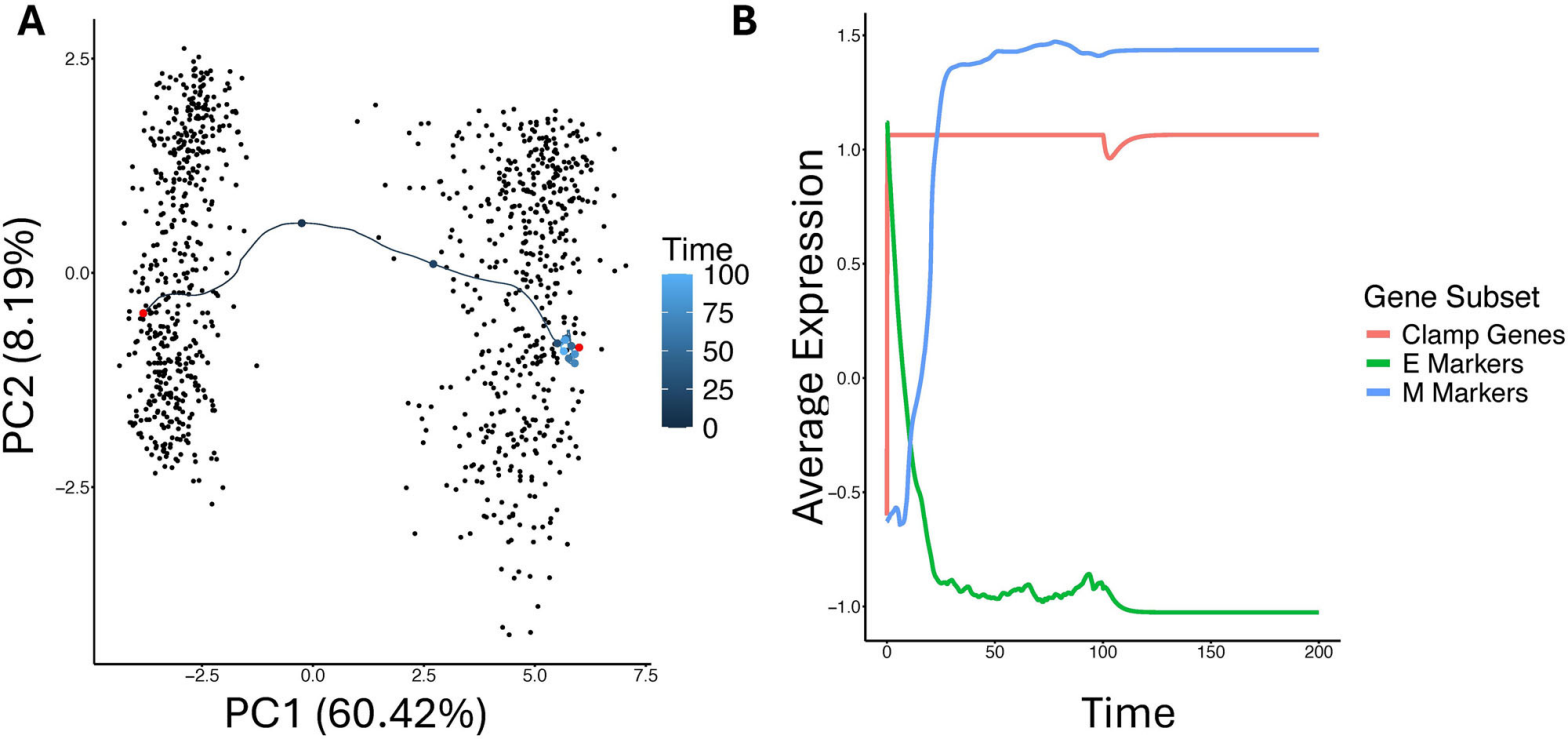
Comparison of Boolean and ODE-based model results. **A** Example trajectory of a model which undergoes a complete EMT under a signal which clamps Zeb1 for 100 time steps, followed by 100 time steps of deterministic simulation with the signal removed. The trajectory is projected to the PCA space from Fig. 4A and time is indicated by color. A circle marks every 10 unit time of the simulation along the trajectory. The two known steady states of the model are indicated by red points. **B** Average gene expression over time from the simulation in (**A**) grouped by E- and M-associated genes (blue and green respectively), through the first 50 unit time. The clamped gene dynamics are shown in red.

Although the differences in model design make it challenging to compare trajectories on a like-for-like basis, we examined simulated trajectories for one specific signaling case involving Zeb1. In the Boolean model, we follow all spin flips during the clamped phase, clamping node 2 (Zeb1) on, with $T=1$, i.e., at fairly low noise. What we find is that most nodes alternate rapidly back and forth. Eliminating these nodes from consideration leaves us with the nodes 4 (Cdh1), 6 (Zeb2), 7 (Snai1), 15 (Snai2), 17 (Twist2), and 20 (Twist1) to track. In all 3 runs we conducted, the first of these to flip is node 6 (Zeb2), followed after a few update cycles, by all the other five in what seems like random order and in quick succession. If we clamp node 6 off, then the transition rate falls from 100% to 1%. Meanwhile, we sampled timepoints from the RACIPE ensemble during EMT driven by clamping Zeb1 and examined the trajectories for models beginning in E states. In these simulations, a one-gene signal affecting Zeb1 was applied and the initial conditions were set to the E-like state for the model, such that the only variation between runs was due to noise. This signal was applied for 100 time units, followed by 100 units of relaxation with no noise or signal. In contrast to the Boolean model, many RACIPE trajectories show strong, immediate responses from several nodes which alternated randomly in the Boolean model, with many models showing relatively direct paths through PCA space from their E-like to M-like states (Fig. 6A). This deterministic destabilization of the E state in some RACIPE models in response to a perturbation affecting Zeb1 was not observed in the single Boolean model. In the aggregate, E-associated genes quickly decrease and M-associated genes show a corresponding increase, with little temporal separation between genes and no apparent distinctive behavior of Zeb2 (Fig. 6B). This discrepancy may be due to the random update order in the Boolean model, whereas the ODE model more natively represents the flow of information along the network edges. Aside from a general trend that genes immediately downstream of Zeb1 in the network tended to show substantial changes earlier on, the trajectories of RACIPE models also varied substantially depending on the parameter set and the positions of the original E and M states.

**Fig. 7:**
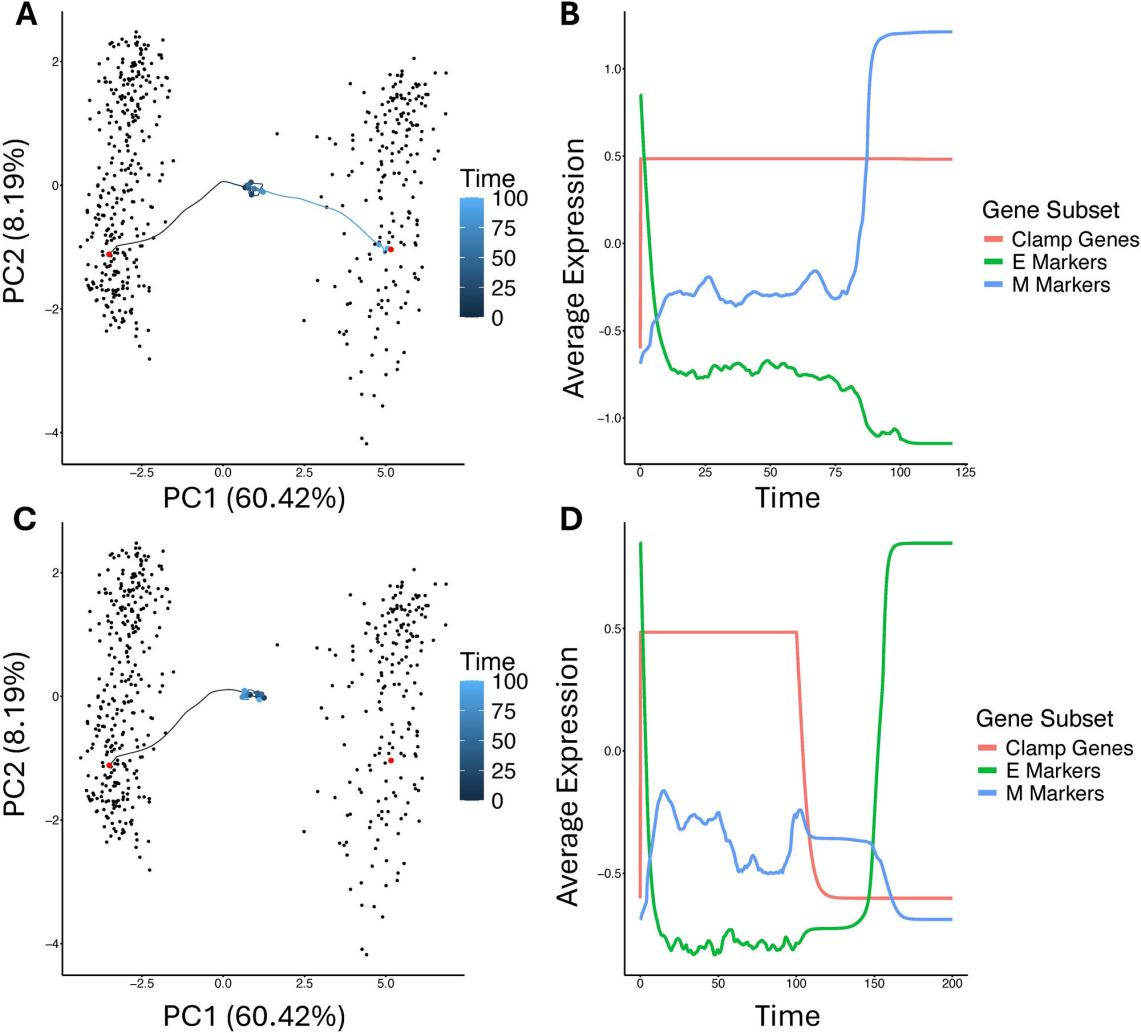
Intermediate states during EMT in ODE-based model. **A** Example trajectory of a model which undergoes a complete EMT under a signal which clamps Zeb1 for 100 unit time, followed by 100 unit time of deterministic simulation with the signal removed. The trajectory is projected to the PCA space from Fig. 4A and time is indicated by color, with circles marking every 10 time units. The two known steady states of the unperturbed model are indicated by red points. **B** Average gene expression over time from the simulation in (**B**) grouped by E- and M-associated genes (blue and green respectively). The clamped gene dynamics are shown in red. **C**, **D** Another instance of the same model being driven by the same signal, in which the model does not undergo complete EMT but lingers in an intermediate state before returning to its initial conditions.

In some cases, the ODE models revealed other behaviors not seen in the Boolean model. Taking another example model, we applied the same signal by clamping Zeb1 and ran several instances of the same simulation. In many cases, the model appeared to move first to an intermediate state. From here, the model continued to perform a complete EMT in some cases (Fig. 7A, B) and in other cases returned to its initial conditions (Fig. 7C, D). Interestingly, these intermediate states appear to be characterized by an intermediate expression of several E- and M-associated genes, a feature of hybrid EMT states previously observed in computational^20^ and experimental studies^23^. To further investigate why so few models transition irreversibly rather than stochastically oscillate between states, we selected a model which produces a new hybrid state under clamping and evaluated the minimum noise threshold to allow transitions in each direction. We simulated the clamped system beginning from each of the steady states possible under clamping: a shifted E-like state (E2), the M-state, and a new hybrid state (H). We observed that the minimum level of noise required to enable transitions to a more M-like state was much lower than the noise which would enable a transition back to a more E-like state (Supplementary Fig. 6). Overall, while the RACIPE transition trajectories appear to depend significantly on the parameter set used, some features such as the order of gene expression changes relative to the clamped nodes’ position in the network and the relative ease of EMT compared to MET were fairly consistent.

## Discussion

By systematically perturbing the EMT transcriptional GRN within two modeling frameworks, a Boolean model and the ODE-based RACIPE framework, we have determined the GRN’s response to a wide range of EMT-inducing signals, finding considerable amplification in signal efficacy when signals are accompanied by sufficient transcriptional noise. Our results highlight the role of noise in facilitating cell state transitions, a property which has been previously observed in the context of EMT and other processes^6^. Generally, across both modeling frameworks, we found that most signals were ineffective under deterministic conditions, but signal efficacy increased substantially when accompanied by transcriptional noise. Zeb1 emerged as the strongest single-gene driver of EMT in both models, likely due to its high out-degree and self-activation, as well as its high betweenness centrality. The RACIPE-based simulations further revealed that the effectiveness of a signal depended not only on network topology but also on initial steady-state properties and parameter heterogeneity, with weak or moderate signals driving transitions preferentially in models already primed for EMT. Trajectories varied across parameter sets, with some models displaying smooth transitions and others passing through intermediate or hybrid states. Together, these results demonstrate that EMT induction can be both signal- and noise-driven, with both GRN topology and cellular heterogeneity shaping the accessibility of phenotypic transitions.

The agreement between the Boolean and ODE-based models at the level of signal efficacy ranking shows that both approaches capture similar network behaviors and provide complementary advantages. The Boolean model has the obvious advantage of computational efficiency and can quickly reveal a coarse-grained picture of the GRN perturbation space. The ensemble of ODE systems incorporates an additional degree of variability through the randomized model parameters, effectively simulating perturbations to a more heterogeneous cellular population. Moreover, the results from both methods are supported by extensive experimental work showing the role of Zeb1 in suppressing expression of Cdh1 and promoting mesenchymal marker genes^24^. The finding that Zeb, Snail and Tgfbeta are more effective drivers of EMT than Twist may be explained by the lower betweenness centrality of Twist1/2 in the network model, and comports with some experimental evidence: in cancer, Twist is associated with collective invasion, and depends on upstream activation of TGF-B to ensure complete EMT^25^. The simulated EMT trajectories also show features observed experimentally, including the possibility of an intermediate third state with moderate expression of miR200^20,23^. However, while the Boolean and RACIPE model may show strong agreement as to the overall state space of the EMT circuit, transition trajectories are not easily predictable from the network topology alone, and may vary substantially according to the framework employed and the particular model's parameters.

The methodology used here is technically applicable to any GRN, though we note that with larger GRNs, including those which might result from statistical inference based on a specific dataset, the computational cost of simulating all pairwise perturbations will grow significantly. Based on our results, in this case we propose limiting the analysis to putative driver perturbations which can be identified by high group betweenness centrality. Based on the overall similarity in results across modeling frameworks, we suggest that some important GRN behaviors are determined by the network topology, largely independent of parameters.

We note that some detailed aspects of the findings such as the rank-ordering of induction signals naturally depend on the input GRN. This is not very surpsing, as different networks possibly reflecting different genetic/epigenetic backgrounds for different cell lines should not be expected to exhibit precisely identical dynamical behavior. This is a lesson that has been learned many times in the EMT community. For example, a high profile paper^26^ focusing on a mouse model for pancreatic cancer claimed that EMT was not needed for metastasis because a knockout of SNA1, of critical importance in many breast cancer contexts, did not affect metastatic spread. It turned out though, that in this specific cancer model, ZEB1 played a much more critical role and a knockout of ZEB1 did indeed interfere with metastasis^27^.

The framework developed here has several limitations. Models may lack additional mechanistic details which make certain signals more or less effective in practice, such as epigenetic regulation, post-translational modification, or other types of regulation not well captured by this set of mathematical assumptions. The parameter range of models comprising the ensemble of ODEs may not accurately reflect the distribution of kinetic parameters in vivo, and some interactions may be incorrect or (more likely) depend on a specific context. Further validation with time-resolved experimental measurements of perturbation response would be necessary to validate the outcomes predicted by the model. In this way, the proposed framework can be useful for quantitatively predicting responses to transcriptional perturbation, creating clear avenues for validation and refinement of the GRN. Applied to a system with a wider range of possible phenotypes, or to an EMT GRN with intermediate states analyzed separately, we anticipate that this method could discern the key drivers of specific pairwise transitions within the landscape. Overall, this approach represents an attempt to systematically probe mathematical GRN models in order to uncover biological design principles and generate testable predictions.

## Methods

### Modeling transcriptional perturbations in the Boolean network model

The Boolean network model is specified by the interaction matrix ${J}_{{ij}}$, giving the influence of node $j$ on node $i$, and by a pseudo-temperature $T$. The ${J}_{{ij}}$ are restricted to the values ±1 and 0. A zero entry indicates no effect, +1 means that node $j$ promotes the expression of node $i$ and a −1 value indicates that node $j$ suppresses the expression. The state of the system at time $t$ is given by the binary spins $\left\{{s}_{i}\left(t\right)\right\}=\pm 1$, with a plus value indicating a high expression level and a negative value a low expression value. The dynamics proceeds by a random update schedule, whereby at each time $\frac{t}{N}$ a node $i$ is chosen at random and the quantity $\Delta E=-2{s}_{i}\left(t\right){{\sum}}_{j}{J}_{{ij}}{s}_{j}\left(t\right)$, the change in pseudo-energy due to flipping ${s}_{i}$, is computed. Then a uniform random variable $r$ between 0 and 1 is drawn. If $r < \mathrm{mi}{\rm{n}}\left(1,{e}^{-\Delta E/T}\right)$, the spin is flipped, i.e. ${s}_{i}\left(t+1\right)=-{s}_{i}\left(t\right)$, otherwise it is left unchanged.

To begin the simulation, we generate a random state of the ${s}_{i}$ and run the dynamics at pseudo-temperature $0 < T\le 4$ until we achieve a low frustration state, with frustration $f\le 10$. The frustration is defined to be the number of unsatisfied links in the state:

1 $$f\equiv \mathop{\sum }\limits_{i,j > i}\theta \left(-{s}_{i}{J}_{i,j}{s}_{j}\right),$$

where $\theta$ is the Heaviside step function. If the epithelial score is greater than 4 (out of a maximum value of +6) we use this final state as the initial state of our transition simulation. Otherwise, we start with a new random state and repeat the above procedure. Once an acceptable starting state is found, we than clamp one or two of the nodes to its mesenchymal value; if the epithelial score of the node is zero, we try both possibilities of clamping and use the one that results in more transitions to a mesenchymal state. We then run the simulation from this clamped state and equilibrate for a time ${t}_{c}$. Afterwards, we remove the clamp and quench the system, setting $T=0.5$, and continue the simulation for an additional time of 10. If the final state has an epithelial score less than -4 we count this as a successful transition.

### Modeling transcriptional perturbations in RACIPE

Steady-state distributions for each topology were simulated with RACIPE, a differential-equation-based software which generates an ensemble of ODE network models with nonlinear dynamics and randomized parameters. The parameters generated by RACIPE include: the maximum production and degradation rates of a target gene $A$, ${G}_{A}$ and ${k}_{A}$ respectively; ${\lambda }_{{B}_{i}A}$, denoting the fold-change of $A$ in response to regulation from TF ${B}_{i}$; ${{B}_{i}A}_{0}$, a threshold of regulator activity determining its effect on a target gene; and Hill coefficient ${n}_{{B}_{i}A}$. The modeling framework is described in full in^28^, but in brief, the dynamics for a gene $A$ regulated by TFs $B$ in RACIPE is given by Eq. 2 below:

2 $$\frac{{dA}}{{dt}}=\frac{{G}_{A}}{{\prod }_{i}{\lambda }_{{B}_{i}A}}{\prod }_{i}\left({\lambda }_{{B}_{i}A}+\frac{\left(1-{\lambda }_{{B}_{i}A}\right)}{1+{\left(\frac{{B}_{i}}{{{B}_{i}A}_{0}}\right)}^{{n}_{{B}_{i}A}}}\right)-{k}_{A}A,$$

Using the EMT GRN as the input topology, ensembles of 1000 (26-node) and 5000 (72-node) randomized parameter sets were created and simulated with 200 randomized initial conditions for each. To simulate the 72-node GRN with greater efficiency, we applied a version of RACIPE implemented in Python^29^, and analyzed the results in R alongside those of the 26-node GRN. To account for cases where noise resulted in gene expression values of 0, steady-state gene expression profiles were log-transformed with a pseudocount of 1 and standardized before all downstream analysis. RACIPE steady states were assigned labels of E (epithelial) and M (mesenchymal) by first clustering the data, then assigning identity based on known EMT genes. In line with previous analysis of this EMT GRN, two clusters are readily apparent in the distribution of steady states^15^. We also confirmed that two was the optimal number of clusters by evaluating the silhouette score for ${k}=\,2$ through 6, which identified 2 as the value that maximized the score. A Gaussian Mixture Model was used to generate cluster labels, via the R package ‘ClusterR’. Cluster identity names were simply assigned based on prior expectation that the cluster with higher average expression of Cdh1 would represent the E phenotype, as this association is well documented. Once each steady state was assigned to a cluster, the original set of models were grouped by the steady states they produced, and a subset of models were selected which were bistable with one state in the E and M clusters respectively. Since full convergence of all trajectories from randomized initial conditions is computationally intensive, we applied a pragmatic filtering approach to efficiently classify models’ steady state distributions. We collapsed near-identical steady states by rounding state variables to two decimal places. From this, we then selected a subset of 500 models (and 556 models in 72-node network) that clearly exhibited bistability, defined as having exactly one stable state in each of the E and M clusters. Extending simulations further may have revealed additional bistable models, but we prioritized models that reliably demonstrated E/M bistability within the available computational constraints. This left ensembles of ~500 models which were used for the subsequent analysis.

Transcriptional perturbations were modeled as follows. First, the RACIPE models were simulated until reaching steady states. These steady states served as initial conditions for the perturbed simulation period, wherein the trajectories of one or more genes were temporarily locked at specific values determined by the following process. First, we determine a gene’s “target value” based on the expression of that gene when the model is in its target state, e.g., to drive a model from E to M using Zeb1, we begin from the E steady state and simulate dynamics when Zeb1 is locked at the value at which it is expressed in the M state. Candidate 1- and 2-gene perturbations were systematically generated in this way and applied to the same initial set of steady states. Beginning from an ensemble of E/M bistable models at steady-state, signal induction was then modeled by locking the value for selected signaling genes at the value at which they are expressed in each model’s target state. This signal implementation was chosen to provide a similar effect to toggling a binary node, while avoiding some inconsistency that may arise from using a constant value to treat the whole diverse ensemble. After a period under signaling, the signal (and noise, if applicable) was removed, i.e., the clamping of values was terminated and the dynamics for the clamped genes were simulated as normal, continuing for another “relaxation” period (Fig. 1B).

For stochastic simulations, noise was modeled as an Ornstein-Uhlenbeck process with a white noise term added for the duration of the signal and is then removed for a secondary relaxation phase. The noise term $U$ at time $t+1$ takes the form of Eq. 3 below, where $D$ represents the variance of the noise, $\tau$ represents the time-correlation, *h* is the time-step and ${z}_{t}$ is a random variable drawn from a Gaussian distribution with mean 0 and standard deviation 1. Unless otherwise indicated, all stochastic simulation results shown were conducted with parameters D = 0.04 and $\tau$ = 10 to create sufficient variability that transitions are possible without entirely eliminating the boundary between states, to improve signal efficacy. The variance of the noise, *D*, is scaled to the expected steady-state expression values of each gene, such that genes with higher average expression will have higher noise and vice versa. At the end of the specified signaling period (t = 500 for this study), both signal and noise (if applicable) are removed, and the ODEs are solved for another 50 time-steps to ensure all models have reached steady-state. After completing simulations, the final steady states were classified according to the original cluster distribution using k-nearest neighbors (KNN) classification with $k=25$.

3 $$U\left(t+h\right)=U\left(t\right)* {e}^{-\frac{h}{\tau }}+D\sqrt{1-{e}^{-\frac{2h}{\tau }}}* {z}_{t},$$

## Supplementary information

Supplementary information

## Data Availability

All work in this study is based on simulation of the GRNs included in the ‘inputs’ directory of the code repository, available at https://github.com/dan-ramirez-23/EMT_Transitions_2025.
