# Supplementary material for: A computational approach for perturbation-induced EMT transitions
Source: NPJ Syst Biol Appl. 2025 Nov 13;11:126. doi: 10.1038/s41540-025-00597-9 (PMC12615684; doi:10.1038/s41540-025-00597-9)
Supplement: Supplementary file 1 — Supplementary information [file 41540_2025_597_MOESM1_ESM.pdf]

Supporting Information

Supplementary Note

The general aspects of the conclusions obtained concerning the 26-node EMT GRN are likely applicable to other gene circuits. To test this, we also evaluated a 72-node EMT GRN. First, this network generated significantly fewer models that were E/M bistable (**Supplementary Figure 7**). After subsetting the E/M bistable models and applying transient perturbations in the same manner as before, we simulated all 1-gene perturbations and the top 150 2-gene perturbations ranked by total out-degree in deterministic and stochastic conditions. Deterministic signals ranged from 0-31% effective for single-gene signals and 0-57% for two-gene signals, with the top driver nodes being nuclear β-catenin (cateninnuc), E-cadherin, Snai1, membrane-bound β-catenin (cateninmemb), and TCF/LEF (**Supplementary Figure 8A**). In stochastic simulations with noise level 0.04, the range of efficacy increased to 21-51% for 1-gene signals and 21-60% for two-gene signals. To measure the effect of noise in general, we examined 16 genes for which the average log-fold change between clusters was smaller than 1e-10, as clamping these genes was effectively equivalent to applying no signal. Based on these simulations, noise was stronger in general for the 72-node network, with the same noise level as in the 26-node network inducing about 20% of models to undergo EMT even in the absence of effective signals.

Much like for the smaller GRN, we observed a strong correlation between the group betweenness centrality of the perturbed nodes and the proportion of models undergoing EMT (**Supplementary Figure 8B**, $\boldsymbol{\rho = 0.91}$) within signals at the same noise level. However, the difference in network structure does affect the ranking of different possible signals. For example, Zeb1 was considerably less effective than in the 26-node GRN, driving only 6% of models to undergo EMT in deterministic simulations and 30% in stochastic simulations. In summary, the results for the 72-node GRN support our general finding that betweenness centrality is indicative of EMT induction strength for a wide range of parameter sets, whereas the rank ordering of specific nodes that emerge as EMT driver genes depends on the input topology. Finally, the basic findings regarding the complementary nature of specific signals and general increase in fluctuation intensity also remain valid for this different network.

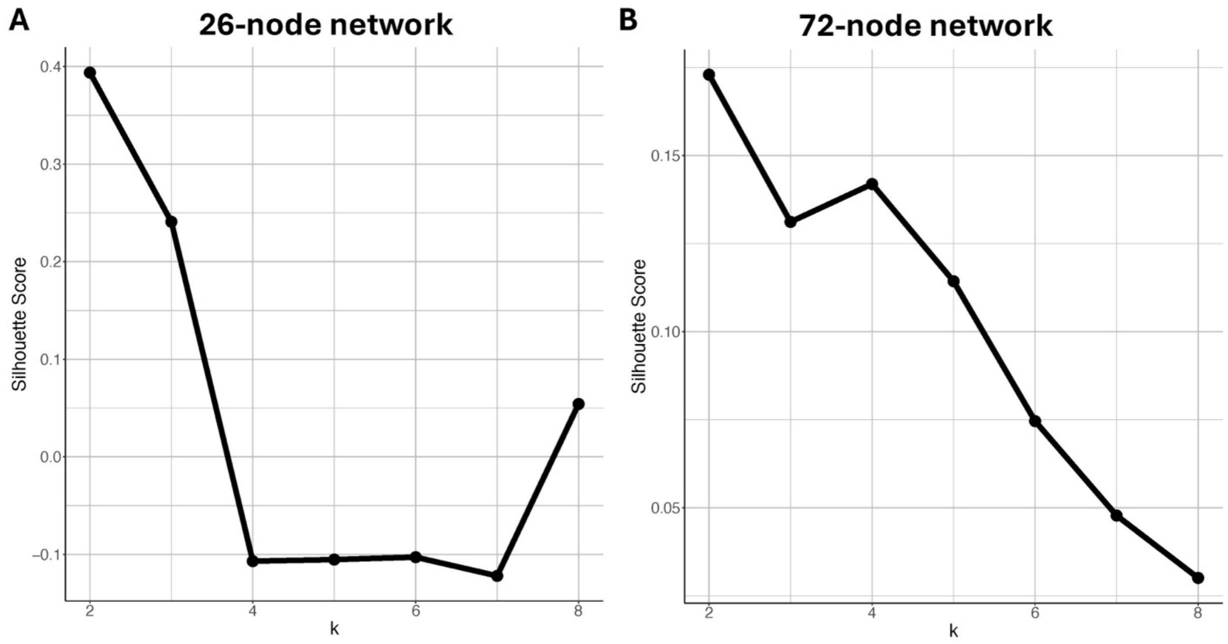

Supplementary Figure 1: Selection of optimal k for clustering unperturbed steady states. A) Line plot showing silhouette score as a function of k, which identifies k = 2 as the optimal number of clusters for the 26-node EMT GRN. **B)** Line plot showing silhouette score vs k for the 72-node EMT GRN. Clustering was performed using a Gaussian Mixture Model applied to the first 15 and 45 principal components for the 26- and 72-node GRNs, respectively.

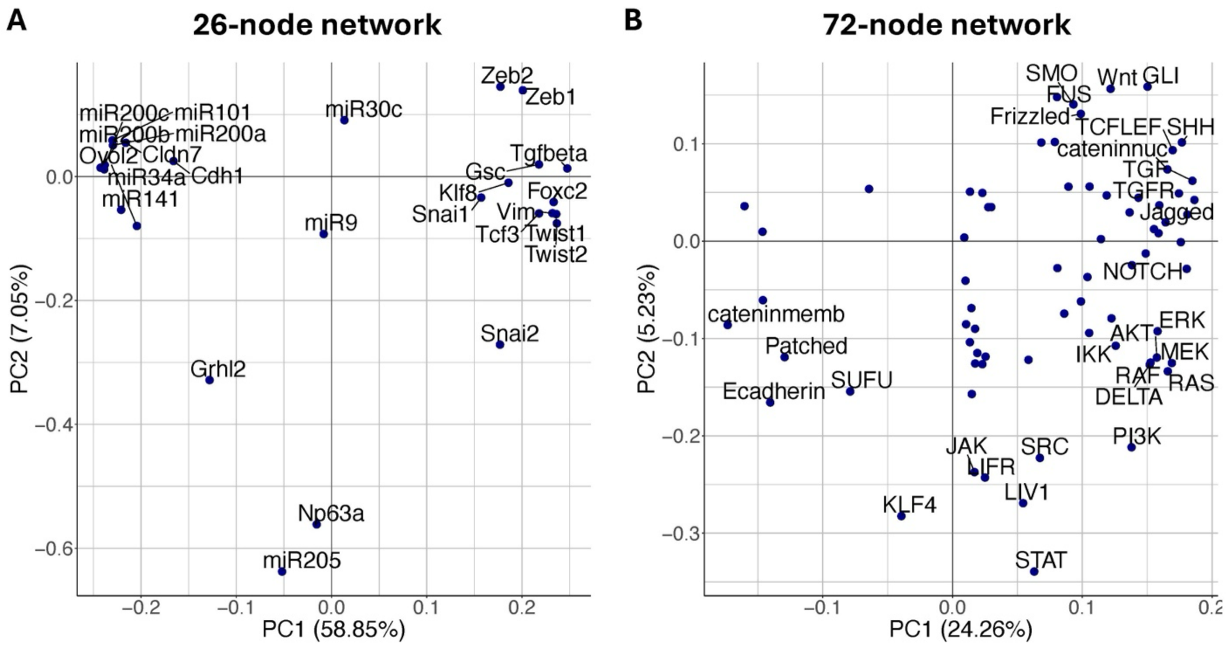

Supplementary Figure 2: Gene loadings for principal component analysis. A) Labeled scatterplot showing the loading positions of each gene in the PCA rotation of the 26-node EMT GRN. **B)** Scatterplot showing the PCA loadings for genes from the 72-node EMT GRN. To limit visual clutter, labels are only drawn for the top 30 genes by contribution.

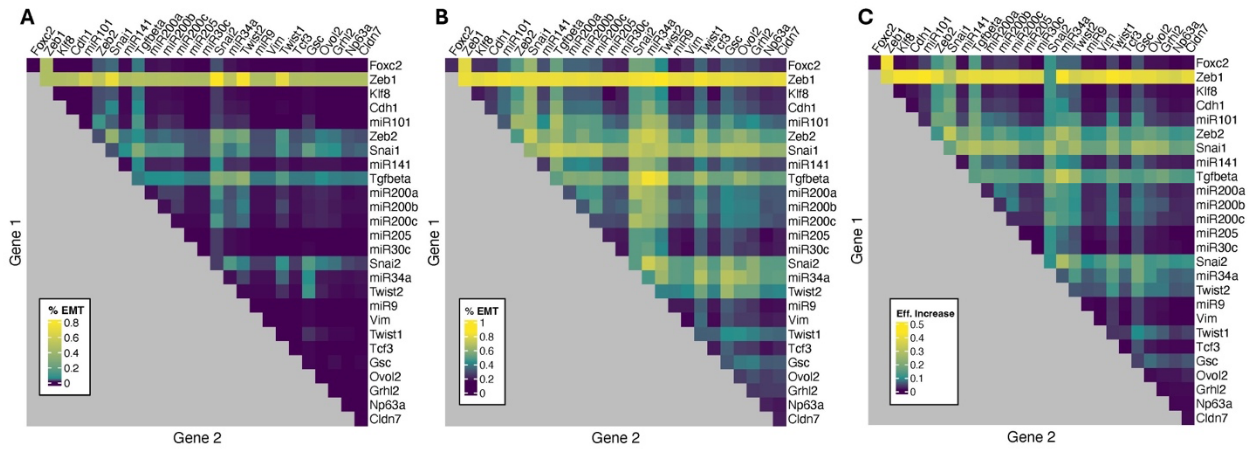

Supplementary Figure 3: Efficacy of EMT-inducing signals in deterministic simulations. A) Heatmap of simulation results for all 1- and 2-node perturbations with no noise applied. Rows and columns denote clamped gene combinations, whereas the color shows the proportion of models which began in an E state and ended in an M state after transient signaling followed by deterministic relaxation. **B)** Same as panel **(A)** for a higher noise level than presented in the main text, with amplitude 0.2. **C)** Effect of noise by signal. Shading denotes the number of additional models undergoing EMT in simulations with noise level 0.04, compared with deterministic simulations, as a proportion of the number of models that resisted EMT for the same signal in deterministic simulations. E.g., if a signal drove 50% of models deterministically, and 75% with noise 0.04, the value in the corresponding cell would be 0.5.

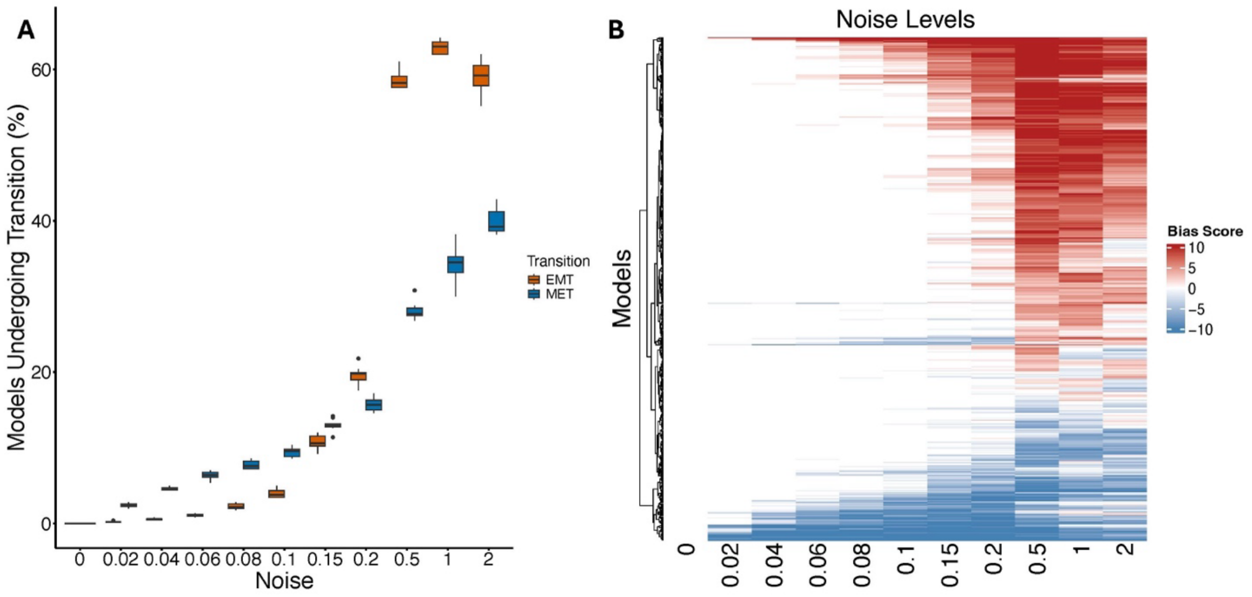

Supplementary Figure 4: EMT/MET driven by transcriptional noise. **A)** Proportion of models undergoing EMT during transient noise simulations with no signal applied. Time correlation for the noise was fixed at 10 and the amplitude varies along the x axis. Boxes show the interquartile range and outliers across a set of 26 trials applied to identical ensembles and simulated for each noise level, followed by relaxation. **B)** Heatmap showing models as rows and noise levels as columns, colored according to a bias score which indicates a tendency of the model to favor EMT/MET in transient noise simulations. Bias score is equal to the number of EMTs observed minus the number of METs observed for a given model over 10 trials; positive scores therefore indicate the model favors EMT, and negative scores favor MET.

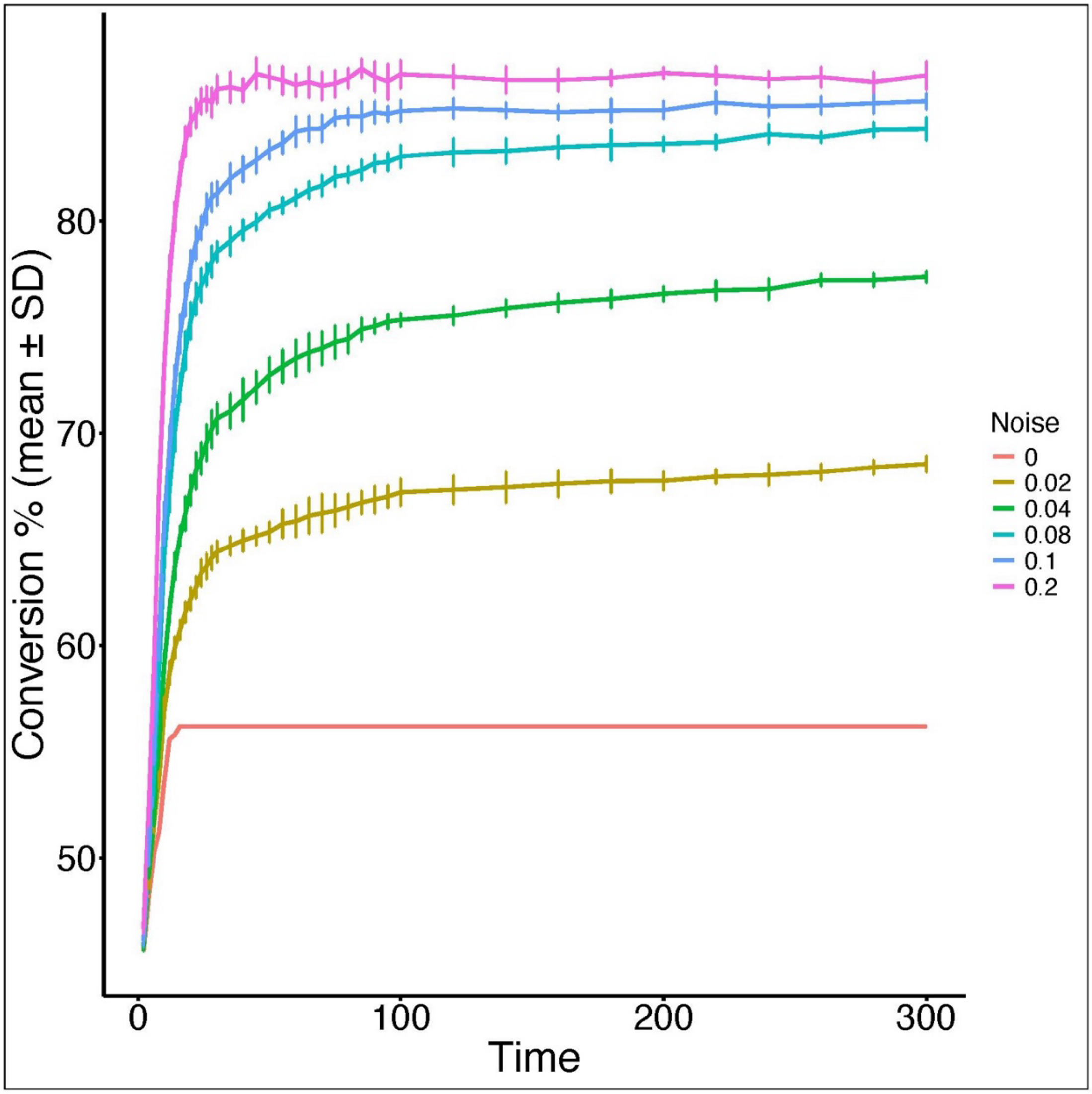

Supplementary Figure 5: Effect of noise on transition time. Results from 10 trials at 6 different noise levels where the same ensemble of 500 E models was given a signal affecting Zeb1 for 300 unit time. Line plots show the mean cumulative percentage of models undergoing EMT over time in each trial, and error bars indicate standard deviation. Noise level is indicated by color.

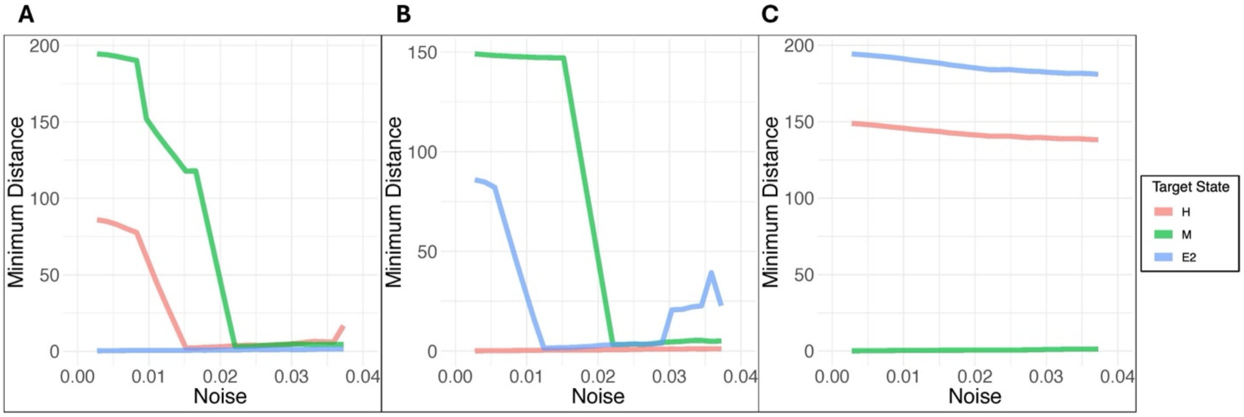

Supplementary Figure 6: Noise thresholds to enable EMT transitions. Beginning from each steady state, we simulated a single model at multiple noise levels for a time of t = 2000, evaluating the minimum Euclidean distance achieved to all other steady states over this time interval. These minimum distances are plotted as a moving average of the nearest 5 noise values. **A)** Minimum distance to each state beginning from an E-like state. **B)** Minimum distance to each steady state beginning from a hybrid state. **C)** Minimum distance to each steady state beginning from an M-like state.

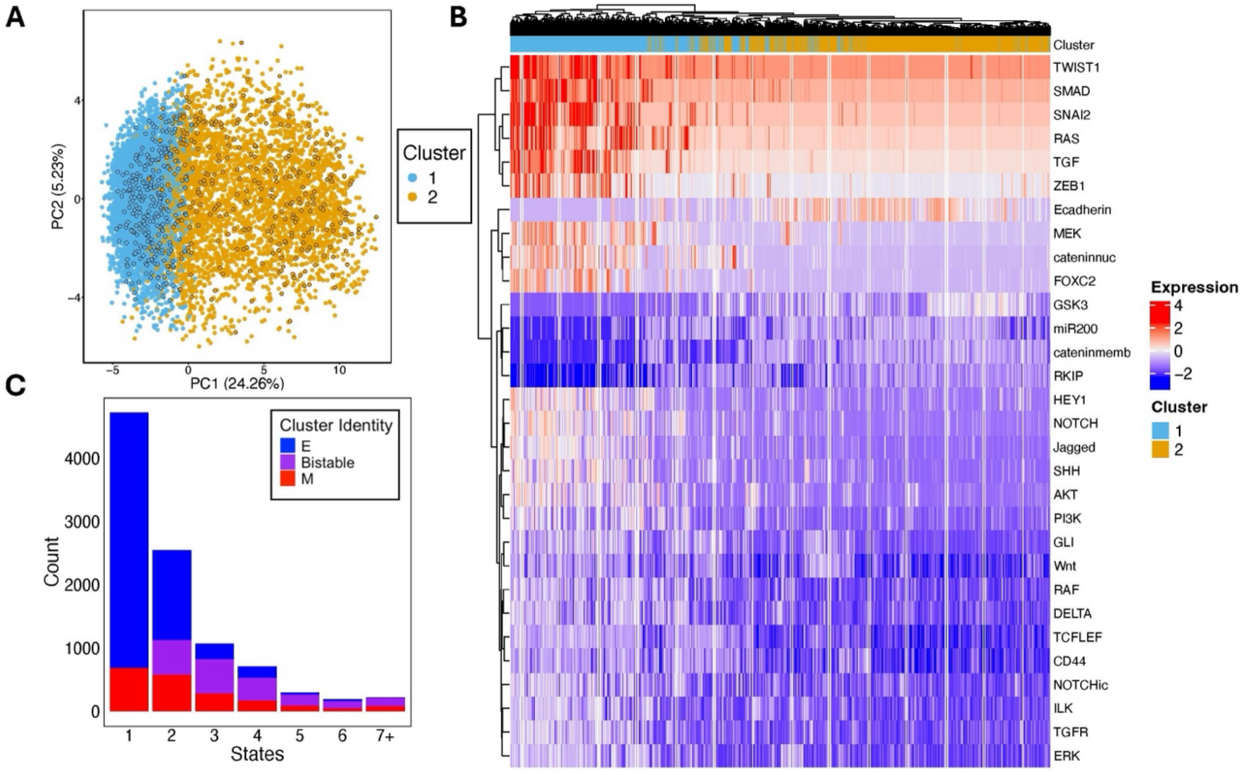

Supplementary Figure 7: Gene expression and multi-stability in the 72-node EMT transcriptional network. A) PCA projection of the unique steady states obtained from simulated models of the 72-node EMT GRN, colored according to labels from Gaussian mixture model clustering. Points with black outline represent states from bistable models selected for further analysis. **B)** Heatmap of gene expression levels across the EMT GRN steady state distribution, annotated on top with cluster labels. Shown are the top 30 DEGs between E and M clusters as identified by limma. **C)** Histogram of the number of unique steady states per model with respect to clusters.

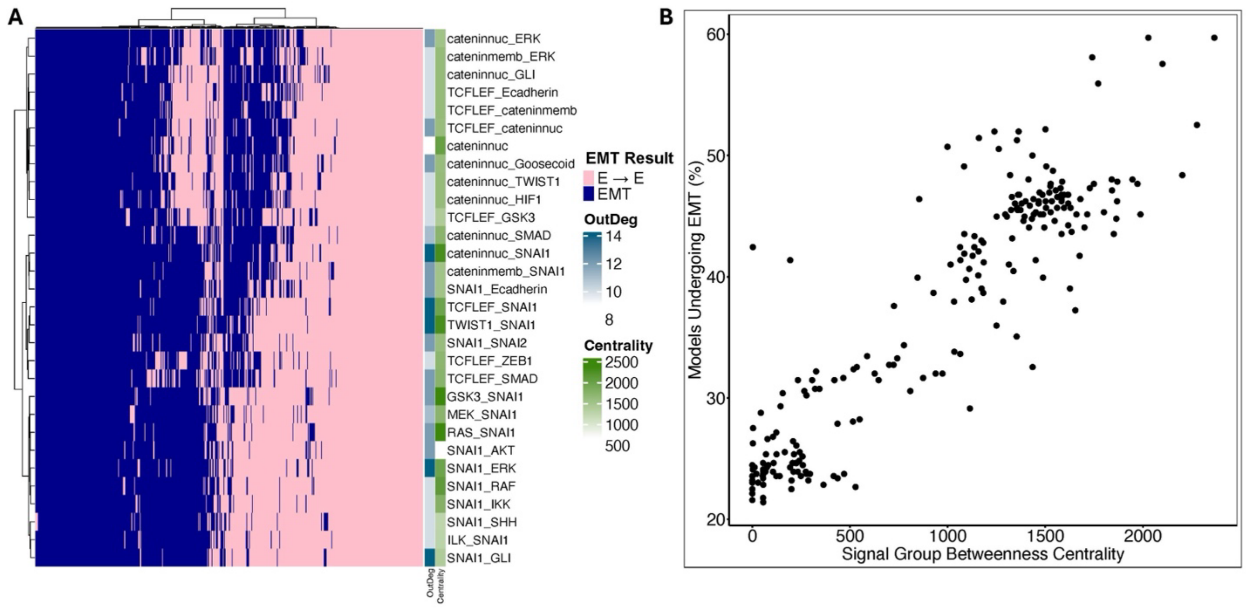

Supplementary Figure 8: Signal efficacy and group betweenness centrality correlate well in the 72-node EMT transcriptional network. A) Heatmap showing the result of signaling simulations by model for the top 30 2-gene perturbations in the 72-node network. Columns indicate models and rows indicate signals. Blue cells indicate models that underwent EMT, while pink cells are models that remained in E states. Signals are annotated by total out-degree (OutDeg) and group betweenness centrality (Centrality). **B)** Scatterplot of results from 72 1-node signals and 150 2-node signals applied to an ensemble of bistable models from the 72-node EMT GRN, with signal efficacy on the y-axis represented as a percentage of models which initiate in the E state and undergo EMT. On the x-axis is the group betweenness centrality of the nodes included in the signal. Simulations were conducted with noise amplitude 0.04 and correlation time 10.

Table S1: Gene name and node number mappings.

Node Number	Gene Symbol
1	Foxc2
2	Zeb1
3	Klf8
4	Cdh1
5	miR101
6	Zeb2
7	Snai1
8	miR141
9	Tgfbeta
10	miR200a
11	miR200b
12	miR200c
13	miR205
14	miR30c
15	Snai2
16	miR34a
17	Twist2
18	miR9
19	Vim
20	Twist1
21	Tcf3
22	Gsc
23	Ovol2
24	Grhl2
25	Np63a
26	Cldn7
